# Effect of nutritional interventions on discharged older patients: study protocol for a randomized controlled trial

**DOI:** 10.1186/s13063-020-04301-6

**Published:** 2020-04-28

**Authors:** Tina Munk, Jonas Anias Svendsen, Anne Wilkens Knudsen, Tanja Bak Østergaard, Anne Marie Beck

**Affiliations:** 1 Dietetic and Nutritional Research Unit, Herlev-Gentofte University Hospital, Herlev, Denmark; 2 University College Copenhagen, Faculty of Health, Institute of Nursing and Nutrition, Copenhagen, Denmark

**Keywords:** Hospital readmissions, Malnutrition, Cross-sectoral transition, Nutritional intervention, Discharge follow-up, Older patients

## Abstract

**Background:**

During hospitalization, many older patients are at nutritional risk or malnourished, and their nutritional condition is often further impaired during hospitalization. After discharge, a “nutrition gap” often occurs in which the patient does not receive enough nutrition to ensure an optimal recovery.

**Methods:**

The study is a randomized controlled study ongoing over 112 days. At discharge, the intervention group receives guidance from a clinical dietitian, and an individualized nutrition plan is made. The dietitian will perform telephone follow-up after 4 and 30 days. It will also be possible for the participant, the participant’s relatives, or the participant’s municipality to contact the dietitian if nutritional questions arise. At the time of discharge, the intervention group will receive a package containing foods and drinks that will cover their nutritional needs on the first day after discharge. They will also receive a goodie bag containing samples of protein-rich, milk-based drinks. Data are collected on quality of life, appetite, physical function, dietary intake, weight, height, energy and protein needs, and experience of discharge and cooperation with the municipality. Information about nutrition status will be sent to the municipality so that the municipality can take over nutritional treatment. The control group receives a standard treatment.

**Discussion:**

This study is the first to combine previously successful single nutritional interventions into a multimodal intervention whose aim is to obtain an effect on patient-related outcomes. We hope that the results will prove beneficial and help to ensure the cross-sector quality of nutritional support to older patients.

**Trial registration:**

ClinicalTrials.gov, NCT03488329. April 5, 2018.

## Background

A multi-international study has shown that, during hospitalization, most older patients are at nutritional risk or malnourished, and their nutritional condition is often further impaired during hospitalization [11]. Malnutrition is directly related to reduced physical function, multiple complications, readmissions, and impaired quality of life [3, 21, 29]. At Herlev Gentofte Hospital, a targeted nutritional effort has been offered to patients at nutritional risk since 2012. The effort consists of a special energy- and dairy protein–enriched food concept consisting of an *a la carte* menu of small dishes enriched with natural energy-dense ingredients and supplemented with a high-quality protein powder and with close follow-up provided by a clinical dietitian. This nutritional effort means that > 85% of the patients with a baseline intake < 50% of the energy and protein requirement achieve ≥ 75% [18]. After discharge, a “nutrition gap” often occurs in which the patient does not receive enough nutrition to ensure an optimal recovery. The lack of nutritional follow-up in connection with older patients’ discharge has a markedly negative effect on functional ability and rehabilitation. This has socioeconomic consequences but also patient-related consequences, such as reduced quality of life and increased dependency on assistance with activities of daily living [15].

According to the recent recommendations of the European Society for Clinical Nutrition and Metabolism (ESPEN), older persons with malnutrition or at risk of malnutrition shall be offered oral nutritional supplements (ONSs) in order to improve dietary intake and body weight and to lower the risk of functional decline after discharge from the hospital [29].

At Herlev Gentofte Hospital and other Danish hospitals, several randomized controlled nutritional intervention studies have aimed at solving the nutritional gap ([1, 2, 6, 9, 14, 24, 25, 27]). These studies have provided individualized dietary counseling, including ONSs if found necessary, but without any provision of food. Even though all these studies have found positive effects on dietary intake and/or nutritional status, the beneficial effect on patient-relevant outcome measures (PROMs), such as quality of life and readmissions, has been very limited ([1, 2, 6, 9, 14, 24, 25, 27]). This is confirmed by systematic reviews conducted in the field [5, 16, 19, 28]. In relation to current national and international guidelines [7, 29], the previous studies may also have been too short in duration to prove beneficial effects on PROMs. In the former studies, the major aim of the dietary counseling has been to improve the intake of energy, and limited focus has been placed on how to optimize the intake of protein to achieve the maximal anabolic response, which seems to be important [12]. In most of the studies, the dietary counseling was provided by dietitians from the hospitals, and only in one study did the municipality play an active role in nutritional support after discharge [27]. Finally, only two of the studies had a focus on dietary intake during the hospital stay [2, 27].

In summary, it appears that in previous studies, single interventions have been examined for a relatively short time and with limited involvement of the municipalities, which may be one explanation for the limited effect on PROMs. The aim of this study is therefore to assess the effect on different PROMs, specifically (re-)admissions, of a long-term multimodal nutritional approach targeted at older patients and involving both hospital and municipality. Our hypothesis is that a more comprehensive intervention study is necessary to elicit a beneficial effect on PROMs.

## Methods

### Study design

**Fig. 1:**
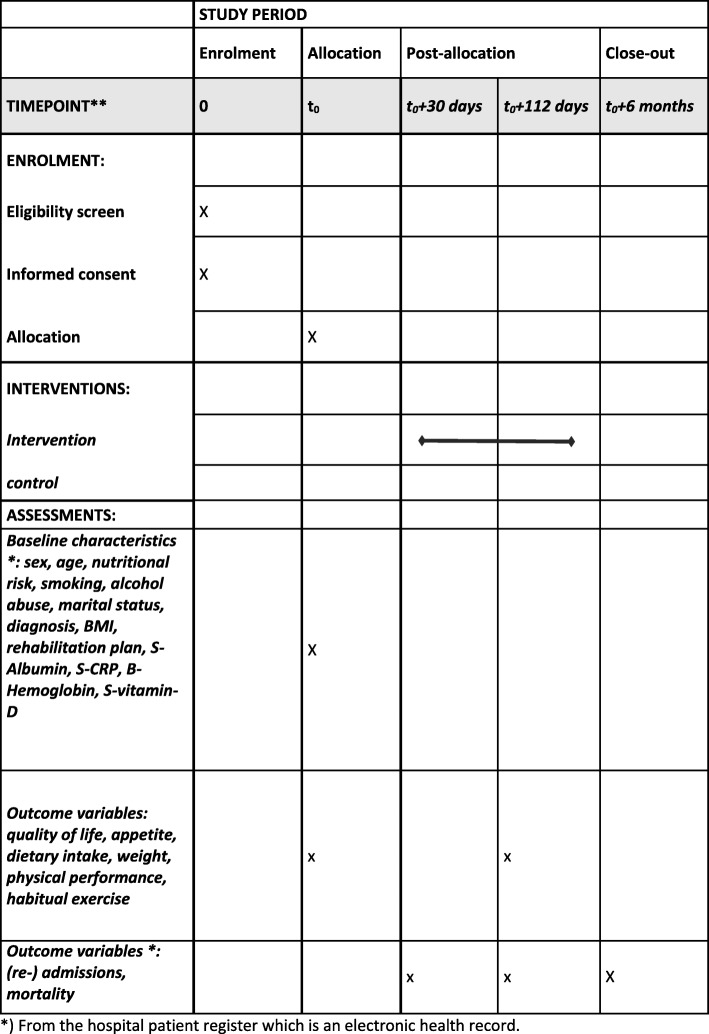
Planned schedule for enrollment, intervention, and assessment in Standard Protocol Items: Recommendations for Interventional Trials format. *From the hospital patient register, which is an electronic health record

The present study protocol describes a 16-week, two-arm, parallel group randomized controlled trial. A planned schedule of enrollment, intervention, and assessment is shown in the Standard Protocol Items: Recommendations for Interventional Trials (SPIRIT) chart in Fig. 1, and items to address in an intervention trial are reflected in Additional file 1: SPIRIT checklist [4]. Flow of the intervention procedure is described in Table 1.

**Table 1:** Multimodal intervention procedure

Intervention group	Baseline, at the hospital	Day 4 after discharge (phone contact)	Day 30 (phone contact)	Day 112
Individualized dietary counseling^a^	x	x	x	x
Individualized nutritional plan^a^	x	x	x	x
Handout nutritional information material^a^	x	X		
Goodie bag^b^	x			
Food package^b^	x			
Communication to municipality^a^	x	(x)	(x)	

^a^Provided by a hospital dietitian

^b^Provided by the hospital kitchen

### Study procedure

#### Recruitment of participants

A sample of 200 (intervention, *n* = 100; control, *n* = 100) patients will be recruited from the oncology, gastrointestinal, and medical departments of Herlev Gentofte Hospital.

##### Inclusion criteria

- 50+ years of age
- In need of preventive nutritional support or in need of nutritional support due to being at nutritional risk according to the Nutrition Risk Screening 2002 tool [13]
- Provided with a targeted nutritional effort consisting of a special energy- and dairy protein–enriched food concept (Herlev’s Glories) with close follow-up of a hospital clinical dietitian [17, 18]
- Discharged to own home (planned)
- Able to read, hear, and understand the Danish language
- Cognitively intact (i.e., not diagnosed with dementia or Alzheimer’s disease)

##### Exclusion criteria

- Food allergy or intolerance
- Planning weight loss or following a special diet
- Receiving enteral or parenteral nutrition at discharge
- Moderate to severe dysphagia, defined as a need for a texture-modified diet
- Permanently bedridden and hence not expected to be discharged to own home
- Discharged to nursing home or rehabilitation stay
- In droplet infection isolation, because not allowed to bring in weighing scales for baseline data collection
- In late palliative or terminal phase, assessed from 6 months of expected survival
- Accelerated and disseminated cancer

There are no restrictions on concomitant care or interventions during the trial, except for those specified in the exclusion criteria.

#### Procedure

Prior to initiation of the study, posters will be developed, and introduction meetings will be held to inform the staff of their role in the recruitment process. Screening and recruitment of study participants will be carried out in collaboration with the departments’ doctors and nurses by the project’s research assistants and research dietitians. The research team will assess interested patients against the eligibility criteria. Individuals deemed eligible and who wish to participate will have their written informed content obtained. Individuals deemed ineligible will have the exclusion criteria recorded. Individuals deemed eligible but who do not want to participate will have their reason for declining recorded and will be asked for permission to use the hospital patient register to collect their baseline and follow-up data recorded there (see Fig. 1).

#### Randomization and blinding

Randomization will take place after each participant has been included in the study, has been assigned an identification code, and has completed the study baseline assessment. The participants will be randomized into two groups: intervention versus control. Before inclusion begins, 28 large, opaque envelopes are created that contain 8 smaller opaque envelopes. The smaller opaque envelopes are sealed and contain a piece of paper stating either “intervention” or “control.” When randomization takes place, the participant selects his or her envelope from the large envelope, thus making it a lottery. When the content of the large envelopes is down to three small envelopes, the small envelopes are transferred to a new large envelope. This is done to prevent situations where the outcome of randomization can be guessed. The large envelopes are kept in a locked cabinet and are only available for the research dietitians and assistants. The hospital dietitians will include participants, and they will gather data at baseline and at 4 and 30 days. A research assistant will collect data at 112 days and 6 months. The research assistant will also oversee data entry. It is not possible to blind involved research assistants, hospital dietitians, and participants, owing to the type of intervention provided. The municipalities will only receive communication regarding the participants in the intervention group and will therefore not know who is in the control group. The statistician will be blind to group allocation until completion of the statistical analysis.

#### Sample size

Power calculations are made relative to the primary endpoint, which is the number of admissions after 6 months. A previous study focusing on a systematic nutritional effort in connection with discharge showed a reduction in the number of admissions (28% in the intervention group and 52% in the control group) during the first 6 months after discharge [2]. By accumulating these numbers with a significance level of 0.05% and a power of 0.80, there must be 64 older patients in each group, a total of 128, to ensure enough power to assess the older medical population. To investigate if patients with cancer also could benefit from the intervention, it is further desired to add a total of 40 extra patients with a cancer diagnosis. However, because it is expected that patients with cancer will be readmitted often, we want to be able to remove these patients from the analyses and still have sufficient power to assess the effect on older medical patients. To also account for dropouts, the goal is 100 patients in each group, for a total of 200 participants.

### Study interventions

The intervention procedure is presented in Table 1 and described in detail below.

#### Intervention group

The intervention group is offered individualized dietary counseling delivered by a research dietitian affiliated with the hospital. The dietitian will perform a comprehensive individualized nutritional assessment to identify the cause of malnutrition, such as lack of appetite, nausea, swallowing problems, polypharmacy, mouth and dental problems, or obstipation. However, the focus is on the dietary intake, activity level, and weight of each participant as a basis for developing a nutrition plan consistent with estimated nutritional requirements and nutritional rehabilitation goals. Specific focus will be placed on optimizing the intake of protein during the day and on the importance of additional strength training. The individualized nutrition plan is delivered together with nutritional information provided on a handout sheet. The counseling will take place in connection with the discharge of the patient. The dietary counseling is standardized so that each session at least contains guidance for meal distribution and meal sizes, counseling in energy- and protein-rich meals and drinks, recommendations for ONSs, a recommendation of protein-rich drinks before bedtime to achieve the maximal anabolic response, the nutrition plan, and recommendation of daily intake of multivitamin tablet.

The individualized nutritional plan, which includes an assessment of nutritional problems, a description of the nutritional therapy started in the hospital, the dietary plan, and finally recommendations for handling the nutritional situation after discharge will be communicated to the municipality (i.e., the general practitioner and the home care if applicable). The purpose of this is that the municipality health care personnel should support the nutritional interventions initiated by the hospital dietitian. If the patient experiences a worsening that requires intervention (e.g., massive weight loss during the intervention period), the municipality will be contacted again.

On day 4 after discharge, the patient will be called by the hospital dietitian for the second individual dietary counseling. The purpose of this is to clarify any additional questions, provide further guidance in the handout nutritional information material, and adjust the nutritional plan if necessary. A need for adjustment of the nutritional plan is based on a questionnaire, where compliance with the nutritional plan, assessment of energy and protein intake in relation to needs, appetite (Simplified Nutritional Appetite Questionnaire [SNAQ]; see “Outcome assessments” section), frequency of strength training, experience of daily diet, use of ONSs, and eventual involvement of home care and startup of home-delivered meals are assessed. Thirty days after discharge, the patient is contacted again by the hospital dietitian for phone-based individual dietary counseling. Again, the purpose is to clarify any additional questions and eventually adjust the nutritional plan on the basis of a questionnaire. The participants and the participants’ relatives are encouraged to contact the hospital dietitian whenever needed and also after day 30.

The intervention group will receive a food package to cover the first 24 h after discharge. The food package is produced by the Nutritional Unit, Herlev Gentofte Hospital, and is based on recipes developed in former projects [17, 22, 23].

The dishes in the food package will consist of energy- and protein-rich main and in-between meals as well as energy- and protein-rich drinks. The food is either naturally rich in energy and protein, enriched with naturally energy-tight ingredients, and/or industrially produced dairy protein powder, when enrichment with natural protein sources is not possible. The daily diet is available in three different energy and protein levels to best cover the patients’ individual needs. The levels of the daily diet start at 6500 kJ and 65 g of protein; the second level is 7500 kJ and 75 g of protein; and the third level is 8500 kJ and 85 g. If needed, the dietitians can further individualize the diet to increase either energy or protein or both.

Food and drink are provided for free to the patient. The first 50 patients in the intervention group will be provided with a questionnaire in which they are asked to note what they think about the food delivered, how the food has been to handle in the home, and what price they might possibly pay for the food delivered.

The intervention group will also receive a free “goodie bag” containing samples of energy- and protein-rich milk products for use in the week after admission. The purpose is for the patients to have the opportunity to taste different flavors. The intervention group will be recommended to take an energy- and protein-rich product just before bedtime for 16 weeks. The participants must purchase these at their own expense. After 16 weeks, the intervention group receives a home visit by research assistants to collect outcome data. A short dietetic counseling session is held if required.

#### Control group

The control group will receive standard care at discharge, which means they follow the standard procedure for discharge, which is different from ward to ward and according to diagnosis. It is the treating ward that decides what standard care entails, such as increased home care or follow-up by the local physician. It does not include dietary counseling or a food package. After 112 days, the control group also receives a home visit to collect data, but no dietetic counseling is performed.

### Outcome assessments

The intervention will last 16 weeks (see Table 1). Assessments of the participants will be conducted by the research dietitian at baseline before randomization. After 16 weeks, data are collected by a research assistant who is not a part of the intervention. In addition, information will be collected from the hospital patient register at baseline and after 4, 30, and 112 days (Fig. 1 shows the SPIRIT figure). Data are collected using specific forms on paper created by the investigators. The researchers will meet regularly to monitor the trial process and check, for example, to see how recruitment is progressing and whether all outcome data are collected according to the protocol to ensure data quality.

Primary and secondary outcomes are described in detail below.

#### Primary outcome

##### Admissions after 6 months

Nonplanned admissions within 6 months will be assessed using data from the hospital patient register, where the number of admissions can be found. The prevalence in each group will be calculated. The length of stay at each admission is also recorded and averaged for the two groups.

#### Secondary outcomes

##### Readmissions

Readmissions, defined as admission within 30 days postdischarge, will be assessed by data from the hospital patient register, where the number can be found. The prevalence in each group will be calculated. The length of stay at each readmission is also recorded and averaged for the two groups.

##### Admissions after 112 days

Nonplanned admissions within the intervention period will be assessed by data from the hospital patient register, where the number of admissions can be found. The prevalence in each group will be calculated and averaged for the two groups.

##### Quality of life at baseline and after 16 weeks

Health-related quality of life will be assessed using the EuroQol–5 dimensions–3 levels (EQ-5D-3L). The EQ-5D-3L questionnaire comprises five domains: mobility, self-care, usual activities, pain/discomfort, and anxiety/depression. Each domain has three levels, ranging from no problems to extreme problems. The raw score must be converted to an EQ-5D-3L score. In addition, the participant is asked to record his/her self-rated health on a vertical visual analogue scale where the endpoints are labeled “Best imaginable health state” and “Worst imaginable health state” [26]. Permission to use the questionnaire has been obtained from the EuroQol Research Foundation.

##### Appetite at baseline and after 16 weeks

Appetite will be assessed using the SNAQ. The SNAQ consists of four questions about appetite, taste, satiety, and number of meals. Each question has five possible answers, and the participants are asked to choose the category that reflects their situation. The questionnaire results in a score ranging from 5 to 20. A score ≤ 14 is predictive of 5–10% weight loss [30]. The score for each participant will be recorded at the two time points.

##### Physical performance status at baseline and after 16 weeks

Physical performance status will be assessed by means of the 30-s chair stand. The participants are asked to fold their arms across their chest and to stand up and sit down on a chair without pushing off with their arms as many times as possible for 30 s. The arms may be used for assistance or for safety if needed [10]. The mode of chair stand will be registered, and the number of chair stands for each participant will be recorded at the two time points. An improvement in physical performance will be considered present if a participant no longer needs the arms when doing the test.

##### Dietary intake at baseline and after 16 weeks

Dietary intake of energy and protein will be assessed by means of a 24-h recall interview. A checklist of specific foods and beverages will be used to verify the reported intake.

Data from the 24-h recall interview will be entered in a dietary program (VITAKOST; https://www.vitakost.dk/da/hjem) that uses data from the national food database. Standard portion sizes from the publication “Dimensions, weight and portion sizes of foods” [31] will be used if insufficient detail is given by the participants. The cutoff for underreporting of energy will be based on an estimated minimum of energy expenditure [8]. The average intake of energy and protein for each participant will be reported and compared with the recommended intake for this population (see below). The results will be presented as a percentage of the requirement.

##### Energy and protein requirements at baseline and after 16 weeks

Energy and protein requirements will be estimated on the basis of a calculation of the basal metabolic rate using the Oxford equations for, respectively, women and men 61–70 and 70+ years of age. To include physical activity, an activity factor of 1.4 is used. Protein requirement is estimated as 1.2 g/kg body wt as recommended for older adults 65+ years of age [20]. The average requirement for each participant will be reported and compared with the dietary intake (see above).

##### Nutritional status at baseline and after 16 weeks

Nutritional status will be assessed by means of weight, which is measured on a calibrated scale with patients wearing light indoor clothes and no shoes. To take the weight of clothes into account, 0.5 kg will be removed from the measured weight. Self-reported information about weight will also be obtained by the research dietitians during the contact with the intervention group. Measurement of height is often not feasible in this old and frail population with chronic disease, because some lack the ability to stand independently. Data on height will be retrieved from self-reported height and only collected at baseline. Body mass index is calculated as actual weight in kilograms divided by the square of height in meters. The average change in kilograms and percentage will be reported for each group.

##### Mortality after 30 days, 112 days, and 6 months

Mortality will be assessed using data from the hospital patient register during the intervention and within 6 months. The prevalence in each group will be calculated.

##### Evaluation of the intervention

As part of the two phone contacts performed by the research dietitians, taste experience of the goodie bag and food package and the experience of the transition between sectors will be assessed. This will be done by means of a questionnaire asking about the appearance and taste of the food and about the experience of the transfer (rated on a scale from 1 to 5, with 5 being best).

### Data and statistical analysis

The full analysis set will follow the intention-to-treat principle and will include all randomized patients for whom a baseline assessment is conducted, regardless of later temporary or permanent loss to follow-up. Lack of outcome data due to loss to follow-up or if data were unobtainable (i.e., if a patient was readmitted to the hospital and did not want to participate any further) will be treated as missing data and will be excluded from the final analysis. Subgroup comparisons will be made between fully compliant participants (i.e., those who, respectively, do and do not achieve an intake of energy and protein at or above 75% of their estimated requirement).

Descriptive and summary statistics will be used to quantify participants’ characteristics and outcome variables. Nonparametric statistics will be applied whenever nonnormality of the outcomes is detected. Unpaired data will be analyzed by Student’s *t* test or the Mann-Whitney *U* test, and paired data will be analyzed by a paired Student’s *t* test or Wilcoxon signed-rank test as appropriate. For dichotomous variables, difference in proportions will be analyzed by a chi-square test. To detect correlations, Pearson’s or Spearman’s correlation will be used as appropriate. For comparison of tests within the group, general linear models will be used. Two-sided tests of statistical significance will be used in statistical analysis. A *p* value < 0.05 will be considered statistically significant. All analyses will be performed using SAS Enterprise guide 7.1 (SAS Institute, Cary, NC, USA).

## Discussion

Several studies investigating single nutritional interventions have previously been conducted and have shown positive effects on nutritional intake and nutritional status. However, effects on PROMs such as quality of life and readmissions are weak. To our knowledge, this study is the first to combine previously successful single nutritional interventions in a multimodal intervention where the aim is to obtain an effect on PROMs.

Such evidence, not least in relation to reduction in expensive admissions, is needed in order to prove the cost-effectiveness of the intervention and hence secure future implementation.

Further, in order to increase the chance of getting the methods implemented in practice after the end of the study, we will try to involve the municipalities more actively in the intervention part of the study, which has not been done in most of the former studies.

Finally, in order to increase the chance of future implementation, we have chosen to broaden our inclusion criteria regarding patient population compared with former studies where the participants consisted of geriatric and orthogeriatric participants.

### Strengths and limitations

A major strength of this study is that we have chosen a longer intervention period than former studies, as also recommended by, for example, ESPEN [29]. This will hopefully increase the chance of finding a positive effect on PROMs.

To our knowledge, this study is the first to combine previously successful single nutritional interventions into a multimodal intervention. A major strength in relation to this is that we have been involved in several of the former single nutritional intervention studies and therefore have detailed information about how these studies were carried out.

Recruitment flow can be difficult when studying older patients at nutritional risk, because they might not be able to overcome participation in the study. Previous studies of older patients have seen a high rate of patients who reject participation. A strength in this study, however, is that we ask participants for permission to collect relevant data from their hospital record in order to investigate if the nonparticipating patients differ significantly from the population receiving the intervention.

Due to the nature of the intervention, it is not possible to blind researchers, dietitians, and participants. However, our primary outcome is risk of admissions, and these data will be collected from the hospital patient register, which will reduce the risk of bias. Furthermore, the statistician will be blinded to group allocation until completion of the statistical analysis, which is also a strength.

One limitation is that we only have close control over the first part of the intervention (i.e., the first 30 days) when the hospital dietitians are involved. Even though the nutritional care plan is communicated to the municipalities and the majority of these have dietitians working with old people, we do not know if these will continue and support the intervention started by the hospital dietitians. Furthermore, we have limited knowledge about the other types of nutritional support available in the municipalities (e.g., energy- and protein-dense meals-on-wheels, nurses in charge of nutrition, availability of the staff to provide support with shopping of recommend foods). Finally, the participants might say “no” to the municipality’s offer. In a former study involving general practitioners, this was an unexpected finding [1].

Also, even though we provide the participants a goodie bag with protein-rich milk products, this is only for 1 week. The rest of the time, the participants are expected to purchase the products themselves, which may not happen.

Today, many Danish hospitals provide patients with a lunch pack at discharge, and some provide ONSs for the first days after discharge. Because of the multimodal design of our study, it will not be possible for us to isolate the beneficial effect of these initiatives. In general, this is also the case with the other individual parts of the multimodal intervention.

Another limitation is that the power calculation is based on a former study including mainly geriatric patients [2], and therefore the risk of admissions may be different in the broader patient population included in the present study. In order to adjust for this, we have increased the number of participants in order to be able to find a reduction in the admission rate within 6 months.

In conclusion, the current status is that older patients found to be at nutritional risk during hospitalization still are at risk at discharge. Studies have shown that organization and communication across sectors is inadequate. Patients are often discharged as soon as possible, when they often still are weak and frail and in need of rehabilitation. The nutritional gap between sectors is often overlooked. These patients need knowledge about what type of food to eat. Furthermore, they need easy access to food right after discharge, as some come home to an empty refrigerator and need help to shop for groceries. The result of the present study will hopefully prove a beneficial effect on different PROMs and help to ensure the cross-sector quality of nutritional support for older patients.

### Trial status

Recruitment of intervention and control group participants was still ongoing at the time of manuscript submission. The ClinicalTrials.gov identifier is NCT03488329, dated April 5, 2018. The date recruitment was completed in October 2019 and the last follow-up data is collected in May 2020.

## Supplementary information

**Additional file 1.** SPIRIT 2013 checklist: recommended items to address in a clinical trial protocol and related documents.

## Data Availability

No data will be made publicly available.

## Abbreviations

EQ-5D-3 L: EuroQol–5 dimensions–3 levels
ESPEN: European Society for Clinical Nutrition and Metabolism
ONS: Oral nutritional supplement
PROMs: Patient-relevant outcome measures
SNAQ: Simplified Nutritional Appetite Questionnaire
